# Barriers to Somatic Health Care for Persons With Severe Mental Illness in Belgium: A Qualitative Study of Patients' and Healthcare Professionals' Perspectives

**DOI:** 10.3389/fpsyt.2021.798530

**Published:** 2022-01-26

**Authors:** Laurence Kohn, Wendy Christiaens, Johan Detraux, Jan De Lepeleire, Marc De Hert, Benoit Gillain, Benjamin Delaunoit, Isabelle Savoye, Patriek Mistiaen, Vicky Jespers

**Affiliations:** ^1^Belgian Health Care Knowledge Centre, Brussels, Belgium; ^2^Department of Neurosciences, Public Health Psychiatry, University Psychiatric Center, Catholic University of Leuven, Kortenberg, Belgium; ^3^Department of Public Health and Primary Care, Catholic University of Leuven, Leuven, Belgium; ^4^Department of Neurosciences, Center for Clinical Psychiatry, University Psychiatric Center, Catholic University of Leuven, Kortenberg, Belgium; ^5^Antwerp Health Law and Ethics Chair, University of Antwerp, Antwerp, Belgium; ^6^Société Royale de Santé Mentale de Belgique, Ottignies, Belgium; ^7^Centre Régional Psychiatrique Les Marronniers, Tournai, Belgium

**Keywords:** physical health, severe mental illness (SMI), health disparities, qualitative research, health care, barriers

## Abstract

**Background:**

A huge and still growing mortality gap between people with severe mental illness (SMI) and the general population exists. Physical illnesses, mainly cardiovascular diseases, substantially contribute to the high mortality rates in patients with SMI. Disparities in somatic health care access, utilisation, and provision contribute to these poor physical health outcomes.

**Methods:**

A qualitative study, using semi-structured interviews, was set up to explore SMI patients' and healthcare professionals' perspectives on somatic health care in different psychiatric settings of the three Belgian regions (Flanders, Brussels, Wallonia). Interviews were digitally recorded and transcribed prior to qualitative inductive thematic analysis, using Nvivo software. The COnsolidated criteria for REporting Qualitative research (COREQ) were used for reporting methods and findings.

**Results:**

Collaboration and information flows between psychiatric healthcare professionals, non-psychiatric healthcare professionals, and persons with SMI were troublesome. This seemed to be mainly due to stigma and prejudice and challenging communication and data transfer. Lack of sufficient training and experience to identify and treat somatic health problems in people with SMI (for psychiatrists and psychiatric nurses) and lack of psychiatric knowledge and feeling or sensitivity for psychiatric patients (for non-psychiatric healthcare professionals) further complicated adequate somatic health care. Finally, optimal somatic follow-up of patients with SMI was hampered by organisational problems (unavailability of equipment, unadapted infrastructure, understaffing, hospital pharmacy issues, and insufficient health promotion/lifestyle interventions), patient-related issues (unawareness of physical problems, non-adherence, need for accompaniment) and financial barriers.

**Conclusion:**

There is an urgent need for integrated somatic and mental healthcare systems and a cultural change. Psychiatrists and primary care providers continue to consider the mental and physical health of their patients as mutually exclusive responsibilities due to a lack of sufficient training and experience, poor or absent liaison links, time constraints and organisational and financial barriers. Modifying these aspects will improve the quality of somatic health care for these vulnerable patients.

## Background

People with severe mental illness (SMI), usually defined as a psychiatric illness that causes serious functional impairment (i.e., schizophrenia, bipolar disorder, or major depressive disorder), have a two to three times higher mortality rate than the general population (1, 2). This increased mortality rate is observed in both high- and low-income countries (1). Somatic comorbidities, mainly cardiovascular diseases, contribute significantly to this excess mortality (3, 4), even in young adults with SMI (5).

Non-medical factors, including an unhealthy lifestyle (high-fat diet, smoking, substance use, lack of physical exercise), and the use of psychotropic medication (particularly antipsychotics) are important risk factors for somatic complications and disorders (2, 6–8). Disparities in somatic health care access, utilisation, and provision may be another cause of the excess mortality due to somatic comorbidities in this vulnerable population. Research has shown that people with SMI often receive fewer physical health screenings and interventions, compared to the general population, even in developed countries (1, 2, 9, 10). Despite clear directions and numerous recommendations over the last decade to improve the quality of somatic health care for people with SMI (1, 10–13), little to no progress has been made. Moreover, it even seems that the mortality gap between people with SMI and the general population is widening (14).

Several patient and illness-, treatment-, healthcare provider-, as well as healthcare system-related factors act as barriers to the recognition and management of somatic comorbidities in patients with SMI (2). A US study (15) showed that lack of awareness of somatic problems, poverty, financial barriers and stigma were primary barriers to oral health care for adult community mental health outpatients with SMI. Cognitive dysfunctions, lack of adherence, lack of integration services, and lack of access to somatic health care have been identified as barriers to appropriate lung cancer (16) and cardiovascular (17, 18) health care among people with SMI. The excess risk of mortality in patients with SMI due to disparities in somatic health, and associated high healthcare costs, make this group of patients an important public health issue that should be addressed (19).

Previous qualitative research (15, 20, 21) indicated that persons with SMI are largely dissatisfied with their somatic health care, due to significant barriers. However, most of this research has been performed in countries with differing healthcare systems from Belgium. The latter is important as Belgium, a country with a population of 11,639,146 (June 2021), has a complex political organisation. It is divided into three highly autonomous regions: Flanders (the Dutch-speaking region in the north), Wallonia (the French-speaking region in the south), and Brussels (the capital, which is officially bilingual). Finally, there is also a minority German-speaking community (in the east of Wallonia). Both federal and regional governments have responsibility for health care in Belgium. The Federal Public Service for Health, Food Chain Safety and the Environment oversees public health care. The regional Flemish, Walloon, and German-speaking communities all have their own administrative healthcare divisions.

## Aim of the Study

The purpose of this study was to identify barriers to somatic health care in the Belgian context by exploring the perspectives on somatic health care of mental healthcare professionals and patients with an SMI in psychiatric settings of different Belgian regions. This study was part of a larger project aimed to examine the status of somatic health care of people with SMI and to understand why this care is sub-optimal in Belgium. Besides exploring the perspectives of patients and healthcare providers on this topic, other aspects (such as the prevalence of somatic problems in people with SMI, and the organisation and financing of somatic health care for people with SMI) have been examined in this project (22). The English version of the full report is accessible on the Belgian Health Care Knowledge Centre (KCE) website. The COnsolidated criteria for REporting Qualitative research (COREQ) were used for reporting methods and findings (see Supplementary Material 1).

## Methods

### Design

The present study applied a qualitative research design. We conducted semi-structured individual face-to-face interviews and group interview sessions with healthcare professionals in several residential psychiatric settings. To explore the patients' perspectives on somatic health care in a psychiatric setting, we planned to conduct focus groups. After concertation with patients associations we thought patients would feel more comfortable in focus groups than during individual face-to-face interviews as they are used to discuss personal issues in groups (e.g., for therapy or in self-help groups). During all (individual and group) interviews, a set of predetermined questions was used to guide the interview. For multidisciplinary healthcare teams and focus groups “case examples – patient scenarios” were used to facilitate the discussion. However, additional questions could be asked where appropriate.

### Settings

For each region (Flanders, Wallonia, and Brussels) we identified four psychiatric settings: 1 psychiatric hospital (PH), 1 general hospital psychiatric ward (GHPW), 1 psychiatric nursing home (PNH) and 1 sheltered housing facility (SHF). Settings were identified through an address list of mental health care settings. We tried to find a balance between private/public, academic/non-academic, and Dutch/French-language settings.

### Participants

#### Patient Inclusion Criteria

Patients were included if they had an SMI (defined in this study as having a diagnosis of schizophrenia or related conditions, bipolar disorder, or moderate to severe depression), for which they had been admitted to one of the four above mentioned psychiatric care facilities.

Patients had to be aged 18 years or older, Dutch or French-speaking, and previously stayed for at least once in the past 5 years in one of the above mentioned types of psychiatric settings. The relatively brief 5 year time period was chosen in order to allow patients to be still able to recall past events fully and accurately.

#### Recruitment Strategy

In January 2020, directors of psychiatric services were contacted personally by telephone. A formal invitation was sent by e-mail, if showing interest (only one director declined to take part in the study due to understaffing problems). Each setting in the sample was visited. During these visits we interviewed: a psychiatrist in an individual face-to-face interview, a somatic practitioner (general practitioner or specialist, if there was no general practitioner entitled to the setting) in an individual face-to-face interview, and the multidisciplinary team (psychiatric nurses, psychologists, educators) in a group interview.

Patients were recruited with the collaboration of patient organisations. They were invited by letter, e-mail, social media, newsletters, or when attending a meeting, and were asked to express their interest to participate in a focus group about their experience with the prevention, treatment, and/or follow-up of their somatic health problems during residential psychiatric care. For this communication, KCE provided a text which was adapted after discussion with and input of the patient organisations. Potential candidates were gathered by the patient organisations, ensuring that the inclusion criteria were met, and a list with the names of the candidates was transmitted to KCE researchers. Next, the KCE researchers contacted the potential participants directly by e-mail, sending the information about the project and the informed consent form (reviewed by one of the patient organisations to ensure readability). The patients were invited to read the information (information about KCE, aim of the study, inclusion criteria, practical information about the study, all necessary information for participation) and informed KCE researchers about their decision to participate or not. The informed consent form was signed before the start of the interview. A moderator read through the information sheet of the informed consent, gave explanations, and answered participants' questions. The moderator also asked permission to audio-record and transcribe the interview.

### Ethical Approval

The qualitative study of the patients' perspectives was submitted and approved by the hospital-faculty ethical comity of the Erasme Hospital (Université Libre de Bruxelles – Belgian Advisory Committee on Bioethics study number CCB B406202042676).

### Data Collection

Based on the literature and exploratory informal meetings with healthcare practitioners, three semi-structured interview guides were developed: one for physicians, one for the multidisciplinary team, and one for patients. Cases describing somatic health problems frequently occurring in the population of psychiatric patients (e.g., weight gain, diabetes) or common acute or chronic problems (a fall, a cough, chronic bronchitis) were used to facilitate the discussion within the multidisciplinary teams. These “case scenarios” were developed and discussed with a representative of one of the patient organisations before finalisation. Based on these scenarios, healthcare professionals were questioned about how somatic health was addressed and managed in their setting. The core topics of the interview guides were:

- What is the place of somatic care in the management of patients: from intake to discharge?
- How is the quality of the management of somatic chronic care perceived?
- What are barriers or challenges in somatic care for chronic and acute health problems, as well as prevention of health problems?
- What are examples of good practises?
- Do you have suggestions to improve somatic health care?
- How is the collaboration between healthcare professionals?

For patients, the same “case scenarios” as for the professionals, were used to structure the discussion if needed. All interviews were moderated in the respondents' native language by KCE researchers. A representative of the patient organisation was present during the interviews or, if not able to attend, contacted the patient after the interview to ensure he/she coped well with the interview and to build trust with the patient. Patients organisations also signed a confidentiality agreement.

Although interviews were originally planned in February, March, and April 2020, due to COVID-19 restrictions, several interviews were postponed to June-July 2020 (for healthcare professionals) and September-October 2020 (for patients). Three (out of 18) individual interviews with healthcare professionals (one with a French-speaking general practitioner and two with Flemish general practitioners) were performed remotely via the online Zoom application. In total, we met about 50 healthcare professionals from 10 different settings (due to the COVID-19 crisis we did not include healthcare professionals for all settings, see section on study limitations). This sample is described in more detail in Table 1. For patients, all focus groups had to be carried out via the Zoom web application with a limit of five participants per session. For each focus group, one KCE moderator was foreseen, accompanied by one observer (a representative of a patient organisation) and one note-taker (KCE researcher). For each region we planned to have two focus groups, each consisting of 6–8 participants. So we intended to meet a minimum of 36 persons with an SMI. However, due to the COVID-19 crisis, the recruitment of participants was hampered. As only four Dutch-speaking patients and for Brussel only one patient finally agreed to participate, focus groups became individual interviews. For Wallonia, five patients were interviewed in one online focus group.

**Table 1:** Description of the healthcare provider participants.

	**Flanders**				**Brussels**				**Wallonia**			
**Type of setting**	**Psychiatric ward**	**Psychiatric hospital**	**Psychiatric nursing home**	**Sheltered housing facility**	**Psychiatric ward**	**Psychiatric hospital**	**Psychiatric nursing home**	**Sheltered housing facility**	**Psychiatric ward**	**Psychiatric hospital**	**Psychiatric nursing home**	**Sheltered housing facility**
Language	Dutch	Dutch	/	Dutch	French	French	Dutch	French	French	French	French	/
Psychiatrist Somatic Physician	1	1	–	1	2	1 + 2^**^	1	1	1	1	–	–
	1 emergency doctor	1 GP	–	1 GP	1 GP	1 GP + 1^*^ GP	1 GP	/	1 GP	1 specialist	1 GP	–
Paramedical team	Team of nurses and psychologist	Team of nurses	/	Team of nurses and educators	/	Team of nurses and social workers	Team of nurses and educators	Multidisciplinary team	Team of nurses, psychologist, physiotherapist, educator	Multidisciplinary team	Multidisciplinary team	/

*GP, general practitioner*.

* *via videoconference*;

** *Psychiatrist in training*.

### Data Analysis

Interviews were audio-recorded. After the interview, a transcript was made by an external firm. Next, the transcripts were coded by two KCE researchers (LK and WC) with NVIVO software. Data were analysed by thematic analysis. An inductive thematic analysis was performed by both researchers. Each researcher made a list of primary codes (WC for the Dutch interviews, LK for the French interviews) without clustering. In a second step, both Dutch and French codes were compared and clustered together, resulting in a hierarchical code tree. Findings were described based on these clusters of codes.

## Results

**Figure 1:**
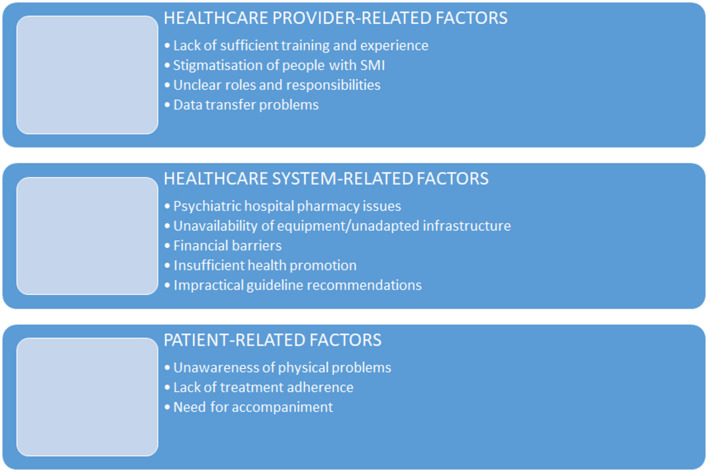
Themes emerging from the qualitative analysis.

An overview of the themes emerging from the qualitative analysis is presented in Figure 1.

### Healthcare Provider-Related Factors

#### Lack of Sufficient Training and Experience

##### Psychiatric Staff

Interviewed psychiatrists referred to their lack of training and experience in addressing somatic health care issues. They explained they were specialised in mental health care already early during their curriculum. Somatic health problems usually were less discussed during training and considered secondary to mental health. In the further course of their career, their knowledge about somatic health care and medical care skills tended to become passive knowledge. Because psychiatrists overemphasised mental health at the expense of somatic health care, they often felt uncomfortable when providing somatic medical care to patients with SMI and rather referred the patient to a general practitioner or specialist for their somatic problems.

“*Whether it's simple hypothyroidism or... a simple lack of vitamins, I think we can handle that. But a patient with severe hypertension, for example, … is still something for an internist or a general practitioner… Our psychiatrists feel uncomfortable when confronted with somatic comorbidities…we have to recognise that we are specialists in psychiatry.” (Psychiatrist-GHPW)*

The same applies to nurses. In PHs or GHPWs, nurses are “psychiatric nurses.” During the interviews, psychiatric nurses mentioned they lost their competencies for a wide range of somatic healthcare tasks such as wound care, injections, or blood sampling. Because they are no longer or less experienced with these tasks, they are often tentative, unsure, or uncomfortable performing them.

##### Somatic Healthcare Providers

Interviewed participants mentioned that transfers to a somatic ward were not self-evident. They stated that staff at the somatic ward seemed very reluctant to take over the patient's care. If patients with SMI were treated at the emergency department, psychiatric staff complained of patients being referred back too soon to the psychiatric ward. Many of these patients did not receive a decent screening of somatic problems. Examinations, like an ECG, were not performed due to the young age of the patient, while the addictive behaviour justified it.

“*If you kindly request an emergency physician in a general hospital to perform an electrocardiogram on a 30-year-old cocaine addict, and he tells you ‘but he is not old enough to have a heart attack', while the patient already had two infarctions...”(Psychiatrist-GHPW)*

Uncomfortable feelings and lack of training and experience to cope with these patients were supposed to be the main reasons for this way of acting by the somatic staff.

##### Patients' Accounts

In general, psychiatric patients themselves experienced the limited somatic skills of healthcare providers as obsolete. They also felt that psychiatric healthcare professionals focus on mental health at the expense of somatic health care.

“*Because my cough persisted for so long without any examination, …well after almost two months of coughing, I insisted that at least a doctor should be called to listen to my lungs at last” (Patient report)*

According to the patients' accounts, the provision of medical care varied substantially from setting to setting (PHs, GHPWs, PNHs, SHFs), within the same setting and among healthcare professionals. Patients also attributed differences in the management of medical care to the type of somatic health problem [priority was given to patients with an addiction or with a known somatic health problem at admission (e.g., diabetes)]. Medical care for unanticipated somatic health needs, however, was problematic. Patients mentioned they were well monitored for adverse drug reactions during the stay, with the exception of weight gain.

#### Stigmatisation of People With SMI

##### Somatic Healthcare Providers

The staff of psychiatric settings reported that dentists, general practitioners, or somatic specialists are less willing to treat residential psychiatric patients than those without such a diagnosis. Psychiatric nurses mentioned that the waiting time could be several days before a specialist arrived. Even for staff within the GHPW, where the care of patients with somatic comorbidities should be easier to manage due to the easy access to any specialisation present within the general hospital, it was difficult to find a specialist willing to come to the GHPW to examine a patient.

According to psychiatric healthcare workers, patients with SMI are often perceived by somatic healthcare providers as non-hygienic, self-neglecting, difficult to understand, non-adherent, skipping appointments, manipulative, attention-seeking, pretending, or they “don't look sick” or “not that sick.”

”*These are patients who... how should I say it, they are not sexy, people tend to be condescending towards these patients, who not always have a neat appearance,.. they don't resemble most patients in a waiting room, or sometimes they are very weird. They talk to themselves, they have, I don't know, weird bags, messy hair. …, they cannot come on time, they come either two hours early or five hours too late, or they come another day… “ (Psychiatrist-GHPW)*

##### Patients' Accounts

A major concern raised by patients was diagnostic misinterpretation or misattribution of signs and symptoms of somatic illness to the SMI, leading to under-diagnosis and mistreatment of the somatic condition, or delayed medical care. Indeed, patients often complained that their somatic health problems were not taken seriously by healthcare professionals. They mentioned that their symptoms were not fully explored or easily misattributed to stress or psychiatric illness. Sometimes healthcare staff even did not listen, ridiculed the patient, did not believe the patient, minimised or denied their problems. In addition, the way they expressed pain or discomfort was often not understood by the staff.

“*Yes, you very often hear other patients on the psychiatric ward say ‘I have something but the doctors don't believe me.' You hear that so often. Or, you go to a hospital and when they see in your medical file that you are admitted to a psychiatric ward, then suddenly they don't take you seriously.”(Patient report)*

Patients also mentioned that some healthcare professionals, even psychiatrists, are patronising.

#### Unclear Roles and Responsibilities

Psychiatric nurses found it very difficult and time-consuming to find out who to consult in case of somatic health problems.

“*The question is often ‘Who does what?'. You have the main treating physician, in this case, the psychiatrist, but does the somatic specialist take over all somatic tasks? Or does he expect us to do certain things ourselves, such as prescribing and adjusting medication, somatic monitoring…”(Team*-*GHPW)*

#### Data Transfer Problems

##### Somatic Healthcare Providers

Healthcare providers stated that at the time of admission to the psychiatric setting the management of existing or chronic somatic problems was often delayed and complicated by the absence of information on the medical history of the patient.

Interviewees reported that data transfer problems occurred frequently and that there was always a risk of losing information.

“*I think of a patient who was very feverish, …, I saw that she had been seen by a general practitioner two hours before, … the note was summarised as ‘hyperthermia, suspected urinary tract infection' and that's all. So we don't know how much temperature, what was done during the physical examination, what was excluded, not excluded. Are there any instructions to follow if the temperature... So it's true …, it would help me a lot if there was more information.” (Psychiatrist in training-PH)*

##### Patients' Accounts

Patients complained about healthcare providers not communicating about the timing of (follow-up) consultations, somatic diagnoses, the prescribed treatment including changes in medication schedule and possible side-effects, and who to contact in case of a somatic health problem during their stay.

### Healthcare System-Related Factors

#### Psychiatric Hospital Pharmacy Issues

Some psychiatric patients with a chronic somatic disease (e.g., diabetes) did not receive the type or brand that was prescribed or which the patient is accustomed to, due to formulary restrictions (i.e., the medication was not available in the formulary or list of medications available for use at the hospital). However, the new medication of choice could be more expensive or less safe (e.g., due to medication compatibility issues during switching) than the restricted agent. A request for formulary addition from the general practitioner was not always granted. Moreover, when a new medicine was prescribed by a general practitioner or a specialist, it took a couple of days to get the prescribed medicine to the patient, because the prescription needed to be approved by the psychiatrist in charge and by the hospital pharmacy.

“*Yes, indeed, if the psychiatrist is present, then you still have to see whether you can reach him to validate that prescription. Then the pharmacy still has to validate the prescription and then it's still possible that they have to order it (…) Yes, so sometimes two days pass before he gets his medication, while across the street in the village, there is a pharmacy and then they have it an hour later. And we need two days…”(Staff-PNH)*

#### Unavailability of Equipment/Unadapted Infrastructure

If somatic treatment following hospitalisation was necessary (e.g., perfusion), it was sometimes difficult to manage within the psychiatric setting due to the unavailability of equipment (e.g., infusion stand) or the lack of adaptive infrastructure (e.g., steps) preventing the patient from moving safely around the ward.

#### Financial Barriers

Often institutional financial constraints were put forward by patients and healthcare professionals as an explanation for inadequate somatic health care. This lack of resources leads to heavy workload as a result of understaffing, insufficient primary care providers (e.g., general practitioners) or non-psychiatric specialists (e.g., dietitian, physiotherapist) in psychiatric settings, the critical shortage of medical equipment, inappropriate infrastructure to provide adequate somatic health care in psychiatric settings, and a nomenclature insufficient to fund appropriate somatic health care by the general practitioner. The current funding also seemed to be insufficient for the general practitioner or specialist to attend team meetings, or to work on electronic medical records.

”*I am a self-employed person, paid on a flat-rate basis, the equivalent of seeing three psychiatric patients per day. In a nursing home, I would see 10 to 15 patients. So obviously, I don't do everything very well. (laughs) (...) In a nursing home, I would earn three to four times more.”(General practitioner-PH)*

#### Insufficient Health Promotion

Several lifestyle intervention and health promotion initiatives were mentioned by the interviewees, such as workshops on health related themes, smoking cessation interventions and the creation of smoke-free environments, interactive toothbrushing education, behavioural weight loss programs, the provision of sport equipment,… However, this seemed not to be a priority in psychiatric settings. Discouraging smoking and encouraging physical activities, for example, seemed to be very challenging due to a lack of time, limited space available for sport activities, specific patient characteristics, and barriers to finance sport facilities or competent technical staff to support sports initiatives or smoking cessation programs in psychiatric facilities. Moreover, patients of GHPWs complained they experience strong barriers to use sports facilities at the general hospital, particularly due to stigma. A domain that was reported to be very difficult to manage (also related to the side effects of the medication), was nutrition. The setting was not always able to supply dietetic food (e.g., in some places residents had to choose between different sugar-sweetened beverages during meals, because mineral water was not available).

#### Impractical Guideline Recommendations

Although recommendations certainly can be very helpful in the acute treatment, prevention and follow-up of somatic health problems, our study indicated that clinical somatic local guidance should be adapted to the specificities of psychiatric patients. Some guidelines were perceived as too general and therefore not applicable to very specific cases and contexts.

### Patient-Related Factors

#### Unawareness of Physical Problems

Interviewed healthcare professionals mentioned that patients had difficulties expressing complaints and accepting that a consultation or examination is necessary. This has several consequences in terms of somatic healthcare provision: longer medical consultations or repetitive consultations for the same complaint. Unfortunately, healthcare professionals are often running out of time.

“*(…) a real psychotic person doesn't know what's up or under and is busy with a lot of other things besides what he feels in his body. They often have no contact or less contact with their bodies. So before you realise that there's something wrong, that something is going on…. And then you still have to take him to the right consultation” (Staff-PNH)*

#### Lack of Treatment Adherence

Interviewed healthcare professionals reported that once treatment is initiated, it is difficult to keep psychiatric patients adherent. Because of their illness, some patients also do not easily accept to be examined or have their parameters taken. According to healthcare providers this leads to a deterioration of somatic problems.

#### Need for Accompaniment

Psychiatric staff reported that psychiatric patients need to be accompanied to somatic health services (e.g., specialist, dentist, emergency service), particularly when the service is external to the psychiatric hospital or ward. Mentioned reasons for accompanying patients on visits to somatic healthcare professionals were: the patient is not calm enough to go alone, runaway risk, long waiting times (becoming a problem in noisy and crowded rooms), and the need to clarify somatic problems. However, accompanying patients weighs heavily on the workload of the healthcare teams, because it is very time-consuming.

Patients noted a less than optimal planning of somatic health care at the time of discharge, and that they were left to their own devices. They had to find a general practitioner outside the hospital, to manage their medication (as there was no supply from the hospital), and to make follow-up appointments for their somatic health care with the general practitioner or specialist.

## Discussion

Our qualitative study showed that healthcare provider-related factors (lack of sufficient training and experience, stigma, unclear roles and responsibilities, data transfer problems), healthcare system-related problems (hospital pharmacy issues, unavailability of equipment/unadapted infrastructure, financial barriers, insufficient health promotion, unadapted recommendations), and patient-related issues (unawareness of physical problems, non-adherence, need for accompaniment) complicates adequate somatic health care.

Emerging evidence shows that well-integrated care can improve the quality of health care and several patient outcomes (23–25). Therefore, healthcare professionals should take a holistic approach to health care for the benefit of the patient (26, 27), and all of the above mentioned barriers to somatic health care should be tackled with this basic idea in mind. For example, information sharing systems within and across different healthcare services, shared protocols between mental and somatic health services, and co-location of services can help solve problems regarding data transfer and unclear roles and responsibilities, and remove barriers to delivering integrated care (28). Being able to access information from single or multiple electronic individual medical records can be an important facilitator, as it allows healthcare professionals to identify and track individuals with an SMI needing somatic health services (28). This, however, requires an adequate IT infrastructure and the tackling of medico-legal barriers. Shared protocols, setting out the responsibilities of mental health and somatic services in delivering somatic health care, is another important facilitator (14, 28). Clear agreements with physicians concerning the somatic health care of patients at the psychiatric ward could also reduce patients' waiting times and anxieties, and improve their medical follow-up.

Nevertheless, a successful ending of this mission requires a certain amount of flexibility and openness on the part of individual healthcare professionals. For example, our study showed that co-location of services does not necessarily lead to better somatic health care for people with SMI. Indeed, somatic and mental healthcare staff should also be willing to collaborate. According to Rodgers et al. (28) this emphasises that people rather than organisational systems or structures are primarily responsible for successful integration of care. In this regard, the concept of liaison services can be very important. Liaison services and care coordinators/navigators certainly can play a pivotal role in improving communication (28). It was noted during the interviews that a liaison person between the specialties (such as general practitioners with a special interest in psychiatry) improved communication and led to better somatic health care. One can also develop policies to promote the use of psychiatric-trained healthcare professionals, such as psychiatric nurses, on somatic wards (29), or vice versa.

Stigmatising attitudes towards people with SMI remain another important barrier to adequate somatic health care (28, 30). Our study showed that somatic healthcare professionals often are hesitant to handle people with SMI, due to prejudices and stigmatisation. Psychiatric staff (including general practitioners) reasoned this might be due to lack of training and experience, feelings of insecurity in dealing with people with SMI, the anticipation that people with SMI are non-adherent, the unkempt presentation of patients, the already heavy workload for somatic healthcare specialists and the complexity and/or the slow pace of working with people with SMI. People with SMI reported diagnostic misinterpretation and patronisingattitudes.

Previous research indeed has shown that non-psychiatric healthcare providers often feel uncomfortable (e.g., feeling anxious) when working with psychiatric patients, due to a lack of essential communication skills, fear of being physically hurt, and stigmatisation and prejudices towards mental illness (29). These negative attitudes can compromise somatic healthcare professionals' ability to respond to medical symptoms and deliver qualitative somatic care (29). Interestingly, several studies (31–33) have demonstrated that even mental healthcare professionals have negative stereotypes and social distance desire towards people with SMI, particularly people with schizophrenia.

Stigmatisation (and somatic care) may be further complicated by patient-related barriers such as cognitive and communication deficits and reduced pain sensitivity. Studies have shown that particularly people with schizophrenia are characterised by a reduced pain sensitivity (partly due to the analgesic properties of antipsychotic medications, partly to hypoalgesia as a potential endophenotype of schizophrenia spectrum disorders) and a decreased ability to communicate pain (due to the cognitive deficits). As people with SMI have high rates of somatic health conditions that are often associated with clinical pain (e.g., diabetes), these painful somatic conditions may often go unreported and lead to delays in the identification and treatment. This contributes to an increased risk of somatic comorbidity and mortality (13, 34, 35).

Some of the above-proposed initiatives can be implemented earlier than others. Effective communication between providers, shared protocols, and the empowerment of individuals to coordinate care needs of people with SMI may be realised rather quickly. The accomplishment of cultural changes and educational innovations to overcome the lack of training in the screening, assessment, and management of somatic health aspects amongst psychiatrists and psychiatric nurses, and vice versa, to reduce negative attitudes towards people with SMI on the part of somatic healthcare professionals by providing them “a guide in the handling of patients with SMI,” and enhance their knowledge about the health risks associated with psychotropic medications, need more time (14).

Clinical experts, consulted for our report (22) repeatedly declared that the integration of primary care providers (in most cases a general practitioner) in psychiatric settings is vital to improving the somatic health care of patients with SMI. Olson et al. (36) showed that the lack of a primary care provider on an inpatient psychiatric ward was associated with increased suffering and poorer overall health in patients with SMI. Despite this, there is a shortage of primary care providers in Belgian psychiatric settings. There are manifold reasons for this: a restricted nomenclature, resulting in general practitioners and somatic specialists being hesitant to provide somatic health care to people with SMI, heavy workload, information-sharing difficulties (not being able to access information from medical records), and difficulties in dealing with the complexity of working with people with SMI. Physicians in our study had a feeling of ambivalence when taking up the somatic health care of these patients. They expressed concern regarding their lack of medical knowledge, limited training, and communication skills in treating mental illness, leading to a lack of confidence and diagnostic misinterpretation.

We also learned from our study that adequate somatic health care is hampered by organisational and logistical issues, such as limited on-site equipment, psychiatric staff time constraints, heavy workload of somatic healthcare professionals, and hospital pharmacy issues.

Healthcare providers in psychiatric settings stated that people with SMI and somatic comorbidities make heavy demands on their available time. They considered the organisation of consultations with somatic specialists not only as challenging but also as time-consuming. Staff members have to arrange the logistics for transport to the external ward or hospital and have to accompany the patient, for example, to ensure he is well-understood by somatic specialists and that follow-up is arranged. These measures, of course, require sufficient staffing. These problems have been confirmed in other studies (37, 38).

Another logistic problem cited by healthcare providers concerned the hospital pharmacy issues. Although formulary restrictions are implemented to reduce drug costs and ensure the appropriate use of pharmaceutical products, they can have negative effects on patient outcomes (particularly medication adherence, clinical outcomes, and treatment satisfaction) and enhance total medical costs by increasing health care resource utilisation (physician visits and hospitalizations) (39, 40).

An important aspect of a holistic approach to health care is to pay attention to the patients' autonomy and self-care behaviours. For example, medication adherence, which in all sections of our full report was identified as a patient- and illness-related barrier (22), has been shown to improve by applying collaborative, patient-centred communications skills (41), even in patients with SMI (42). However, the benefits of achieving patient-centred care for medication adherence through techniques such as motivational interviewing and shared decision making in people with SMI are minimal and less conclusive than in general medicine (43–45). Nevertheless, the success of these techniques may be improved if the relationship between patient and therapist is trusting and the technique is adapted to the patient's process and values (46).

Finally, healthcare professionals should focus not only on the screening and acute management of physical health aspects in people with an SMI, but also on the prevention and follow-up of patient's somatic health problems (47). Research (10, 48, 49) has shown that the integration of team members trained in a non-psychiatric discipline (e.g., nutrition, physiotherapy), and the involvement of peers, family, or volunteers to support people with SMI in making lifestyle behaviour changes or healthcare choices, improves their somatic health care. Lifestyle behaviour interventions, such as smoking cessation interventions, behavioural weight loss programmes, and psychoeducation (combined with behavioural interventions) are effective for persons with an SMI. Peer-led programmes for self-management of comorbid general medical conditions are effective for improving the health status of patients with an SMI (e.g., physical health-related quality of life, medication adherence) and the utilisation of healthcare services by these patients (50, 51). An ongoing randomised controlled trial is investigating the feasibility of a novel intervention involving training volunteers to be 'Health Champions' to support people with SMI using mental health services to manage and improve their physical health (52). Follow-up after discharge from psychiatric hospital is another necessity. After residential psychiatric care, general practitioners should be actively implicated by psychiatrists in providing post-discharge care of patients.

## Limitations of the Study

Due to the COVID-19 crisis, we were not able to recruit as many participants as planned. Consequently, we did not reach data sufficiency. Moreover, we were obliged to “meet” the participants online. This way of data gathering may have resulted in a selection bias: patients had to feel comfortable with the use of information technology and the “distant” communication imposed by the video conference. It is therefore probable that we met patients with a higher socioeconomic status than would have been the case if we had been able to recruit people for an “in person” face-to-face interview. From the researchers' point of view, it is more difficult to create an atmosphere of trust and empathy in an online interview. Patients were also recruited through patients' associations. The associations might represent a specific type of patient, being involved in and aware of “self-care.” On the other hand, one could also argue that given the inclusion of patients probably having a better somatic health status, our results may be rather conservative, having missed the most poignant storeys. All this means that our findings are not generalisable to all psychiatric settings and are in fact hypotheses that necessitate further research to come to firm conclusions. In addition, as a general observation we would like to emphasise the large variation we found in patients' accounts. Apart from individual differences, also organisational settings largely diverged. However, due to the small number of participants, we could not do specific subgroup analyses. In other words we could not differentiate between GHPWs, PHs, PNHs, and SHFs. We were forced to draw up general conclusions, without specifying the setting. In addition, during the interviews most attention was paid to what went wrong, leaving positive accounts largely unaddressed. However, this does not mean positive experiences are non-existent.

## Conclusion

There is an urgent need for integrated somatic and mental healthcare systems and a cultural change. However, integrated care for people with SMI and somatic comorbidities is still a long way from becoming a reality. Psychiatrists and primary care providers continue to consider the mental and physical health of their patients as mutually exclusive responsibilities. Lack of sufficient training and experience, poor or absent liaison links, time constraints and organisational and financial barriers, limit the ability of most healthcare professionals to focus beyond their specialty. Modifying these aspects will improve the quality of somatic health care for these vulnerable patients. However, above all, a certain amount of flexibility and openness, as well as a willingness to communicate on the part of individual healthcare professionals is a prerequisite for successful management of somatic health care barriers.

## Data Availability Statement

The raw data supporting the conclusions of this article will be made available by the authors, without undue reservation.

## Ethics Statement

The studies involving human participants were reviewed and approved by Ethical Committee of the Erasme Hospital (Université Libre de Bruxelles – Belgian Advisory Committee on Bioethics Study Number CCB B406202042676). The patients/participants provided their written informed consent to participate in this study.

## Author Contributions

All authors listed have made a substantial, direct, and intellectual contribution to the work and approved it for publication.

## Funding

This work was funded by KCE, Belgian government.

## Conflict of Interest

The authors declare that the research was conducted in the absence of any commercial or financial relationships that could be construed as a potential conflict of interest.

## Publisher's Note

All claims expressed in this article are solely those of the authors and do not necessarily represent those of their affiliated organizations, or those of the publisher, the editors and the reviewers. Any product that may be evaluated in this article, or claim that may be made by its manufacturer, is not guaranteed or endorsed by the publisher.
